# Highly expressed SLCO1B3 inhibits the occurrence and development of breast cancer and can be used as a clinical indicator of prognosis

**DOI:** 10.1038/s41598-020-80152-0

**Published:** 2021-01-12

**Authors:** Tiantian Tang, Guiying Wang, Sihua Liu, Zhaoxue Zhang, Chen Liu, Fang Li, Xudi Liu, Lingjiao Meng, Huichai Yang, Chunxiao Li, Meixiang Sang, Lianmei Zhao

**Affiliations:** 1 Breast Cancer Center, The Fourth Hospital of Hebei Medical University, Shijiazhuang, 050035 Hebei Province China; 2 Department of General Surgery, The Fourth Hospital of Hebei Medical University, Shijiazhuang, 050035 Hebei Province China; 3 Department of General Surgery, The Third Hospital of Hebei Medical University, Shijiazhuang, 050001 Hebei Province China; 4 Research Center, The Fourth Hospital of Hebei Medical University, Shijiazhuang, 050035 Hebei Province China; 5 Department of Pathology, The Fourth Hospital of Hebei Medical University, Shijiazhuang, 050035 Hebei Province China

**Keywords:** Breast cancer, Cancer, Molecular medicine

## Abstract

The role of organic anion transporting polypeptide 1B3 (SLCO1B3) in breast cancer is still controversial. The clinical immunohistochemical results showed that a greater proportion of patients with negative lymph nodes, AJCC stage I, and histological grade 1 (*P* < 0.05) was positively correlated with stronger expression of SLCO1B3, and DFS and OS were also increased significantly in these patients (*P* = 0.041, *P* = 0.001). Further subgroup analysis showed that DFS and OS were significantly enhanced with the increased expression of SLCO1B3 in the ER positive subgroup. The cellular function assay showed that the ability of cell proliferation, migration and invasion was significantly enhanced after knockdown of SLCO1B3 expression in breast cancer cell lines. In contrast, the ability of cell proliferation, migration and invasion was significantly reduced after overexpress the SLCO1B3 in breast cancer cell lines (*P* < 0.05). Overexpression or knockdown of SLCO1B3 had no effect on the apoptotic ability of breast cancer cells. High level of SLCO1B3 expression can inhibit the proliferation, invasion and migration of breast cancer cells, leading to better prognosis of patients. The role of SLCO1B3 in breast cancer may be related to estrogen. SLCO1B3 will become a potential biomarker for breast cancer diagnosis and prognosis assessment.

## Introduction

Breast cancer is one of the most common malignant tumors in women, and its incidence is increasing annually. The incidence of breast cancer in China has risen to number one among female malignancies. Studies have shown that genetic mutations are involved in the occurrence and development of breast cancer, and may imply a role in the prognosis of breast cancer^1^.

Solute carriers (solute carriers, SLC) are the main family of transporters involved in drug uptake, and play important role in determining the efficacy (sensitivity and resistance) and toxicity of chemotherapy^2^. Family member SLCO1B3, also known as organic anion transporting polypeptide 1B3 (OATP1B3)^3^, LST-2^4^, or OATP8^5^, is mainly expressed in the basement membrane of liver cells around the central vein. Its main function is to transport a variety of internal and external substances into liver cells for metabolism^6^. Nagai et al.^3^ first reported that SLCO1B3 is expressed in colon and lung cancer tissues, but not in normal tissues. Later studies showed that SLCO1B3 is also highly expressed in prostate, pancreatic, and ovarian cancer, but the different expression levels are not correlated with prognosis in different cancer types^4–7^. The previous studies also investigated the role of SLCO1B3 in breast cancer, but there was no unified conclusion^8,9^. The expression and function of SCLO1B3 in breast cancer are also not clear. This study mainly explored the relationship between SLCO1B3 and breast cancer, and provided more in vitro evidence to illustrate the role of SCLO1B3 in the development, invasion, and metastasis of breast cancer.

## Results

### The relationship between SLCO1B3 expression and the clinicopathological characteristics

The results showed that the expression of SLCO1B3 was associated with lymph node metastasis, histological grade, AJCC stage and Ki-67 (*P* < 0.05). The higher the expression level, the less the lymph node metastasis, and the higher the expression level, the lower the histological grade and AJCC stage according to Pearson correlation test (*P* < 0.05) (Table 1).

**Table 1:** Different SLCO1B3 expression e.

	Number (n)	Negative SLCO1B3 n (%)	Moderate SLCO1B3 n (%)	Strong SLCO1B3 n (%)	Χ^2^	*H*	Pearson	Pearson
						*P*		*P*
**Age at diagnosis (years)**								
≤ 35	21	5 (23.8)	12 (57.1)	4 (19.0)	0.436	0.509		
> 35	294	57 (19.4)	165 (56.1)	72 (24.5)				
**Menopausal status**								
Pre	154	29 (18.8)	92 (59.7)	33 (21.4)	0.235	0.628		
Post	161	33 (20.5)	85 (52.8)	42 (26.7)				
**Tumor size (cm)**					0.464	0.496		
≤ 2	157	32 (20.4)	80 (51.0)	45 (28.7)				
> 2, ≤ 5	151	30 (19.9)	92 (60.9)	29 (19.2)				
> 5	7	0 (0)	5 (71.4)	2 (28.6)				
**AJCC stage**					9.805	0.002	− 0.177	0.002
I	91	15 (16.5)	41 (45.1)	35 (38.5)	11.237	0.004*		
II	156	29 (18.6)	97 (62.2)	30 (19.2)	1.495	0.474**		
III	68	18 (26.5)	39 (57.4)	11 (16.2)	2.851	0.240***		
**ER**					1.080	0.583		
Positive	224	42 (18.8)	130 (58.0)	52 (23.2)				
Negative	91	20 (22.0)	47 (51.6)	24 (26.4)				
**PR**					0.003	0.960		
Positive	196	36 (18.4)	115 (58.7)	45 (23.0)				
Negative	119	26 (21.8)	62 (52.1)	31 (26.1)				
**HER-2**					22.798	< 0.001	− 0.088	0.118
Positive	176	24 (13.6)	93 (52.8)	59 (33.5)				
Negative	139	38 (27.3)	84 (60.4)	17 (12.2)				
**Molecular subtype**					0.305	0.581		
Luminal A	32	4 (12.9)	24 (77.4)	3 (9.7)				
Luminal B	148	31 (20.9)	80 (54.1)	37 (25.0)				
HER-2 type	77	10 (13.0)	53 (68.7)	14 (25.6)				
TNBC	31	11 (18.8)	13 (56.3)	7 (25.0)				
**LN metastasis**								
Negative	167	30 (18.0)	84 (50.3)	53 (31.7)	11.259	0.004		
Positive	148	32 (21.6)	93 (62.8)	23 (15.5)				
**LN metastasis**					6.942	0.008	− 0.149	0.008
0	167	30 (18.0)	84 (50.3)	53 (31.7)	6.863	0.032*		
1 ~ 3	85	17 (20.0)	54 (63.5)	14 (16.5)	0.369	0.831**		
> 3	63	15 (23.8)	39 (61.9)	9 (14.3)	7.119	0.028***		
**Histological grade**					4.961	0.026	− 0.126	0.026
1	9	1 (11.1)	3 (33.3)	5 (55.6)	4.481	0.106*		
2	255	47 (18.4)	146 (57.3)	62 (24.3)	2.590	0.274**		
3	51	14 (27.5)	28 (54.9)	9 (17.6)	6.217	0.045***		
**Ki-67**								
< 14%	245	50 (20.4)	139 (56.7)	56 (22.9)	1.090	0.580		
≥ 14%	70	12 (17.1)	38 (54.3)	20 (28.6)				
**Vascular invasion**								
Yes	257	52 (20.2)	143 (55.6)	62 (24.1)	0.098	0.755		
No	58	10 (17.2)	34 (58.6)	14 (24.1)				
**Chemotherapy**					5.968	0.051		
No	151	37 (24.5)	75 (49.7)	39 (25.8)				
Yes	164	25 (15.2)	102 (62.2)	37 (22.6)				
**Endocrine therapy**					5.329	0.070		
No	254	52 (20.5)	135 (53.1)	39 (26.4)				
Yes	61	10 (16.4)	42 (68.9)	37 (14.8)				

Pairwise comparison between *I vs. II, 0 vs. 1 ~ 3, 1 vs. 2, **II vs. III, 1 ~ 3 vs. > 3, 2 vs. 3, ***I vs. III, 0 vs. > 3, 2 vs. 3. P values less than 0.05 are considered as significant changes.

### The relationship between SLCO1B3 expression and breast cancer prognosis

#### The relationship between SLCO1B3 expression and DFS/OS

**Figure 1:**
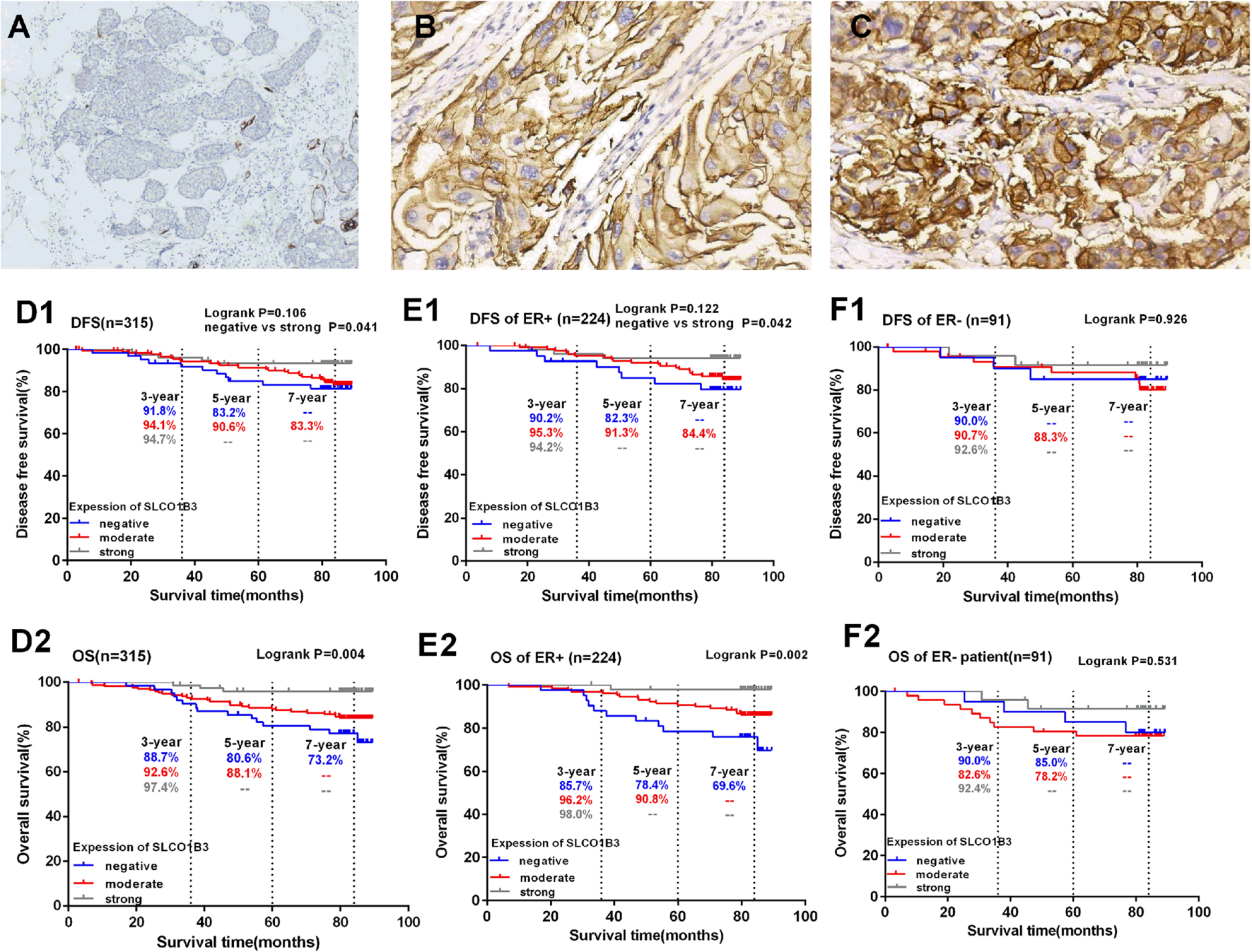
SLCO1B3 staining in representative tissue specimens, and the association between SLCO1B3 expression and the clinical outcome of patients with primary invasive breast cancer. The immunohistochemistry staining of SLCO1B3 on representative tumor specimens and normal breast tissue are shown. Panel (**A**) shows the negative SLCO1B3 expression in breast cancer tissue (× 200); panel (**B**,**C**) show moderately positive (**B**) and strongly positive (**C**) SLCO1B3 expression in the breast cancer tissues (× 200). Kaplan–Meier curves for DFS and OS were plotted based on different SLCO1B3 expression levels in all breast cancer patients **(D1**,**D2**), or in ER + breast cancer patients (**E1**,**E2**) and ER- breast cancer patients (**F1**,**F2**).

The follow-up ended in June 2019, with a median follow-up time of 77.2 ± 17.6 months, ranging from 3.1 to 89.5 months. There was no significant difference in the KM survival curves of DFS among the three groups (*P* = 0.106), the strongly positive group had significantly increased DFS than the negative group after pairwise comparison (*P* = 0.041). The OS showed a trend that the higher the expression of SLCO1B3, the better the OS (*P* < 0.05) (Fig. 1D1,D2).

We also conducted a stratified analysis based on ER expression. The results showed that in the ER positive patients, there was no significant difference in DFS among the three SLCO1B3 expression groups (*P* = 0.122), but the pairwise comparison showed that the strongly SLCO1B3 positive group had significantly higher DFS than the negative group (*P* = 0.042). The OS showed a trend that the higher the expression of SLCO1B3, the better the OS (*P* = 0.002) (Fig. 1E1,E2). However, in the ER negative patients, there was no association between DFS/OS and SLCO1B3 expression level (*P* = 0.926, *P* = 0.531), even between the negative group and strongly positive group (*P* = 0.500 for DFS, *P* = 0.303 for OS) (Fig. 1F1,F2).

To further validate the prognostic value of SLCO1B3 expression for breast cancer patients, the Kaplan–Meier plotter database (https://kmplot.com/analysis/) was searched and analyzed. The results indicated that the median survival of patients with high SLCO1B3 mRNA expression were longer than that of those with low mRNA expression (median OS: 216.66 vs. 185.16 months), but the difference did not reach a statistical significance (*P* = 0.076) (Fig. S1). In contrast, there was a significant relationship between high SLCO1B3 expression and better survival outcome after excluding breast cancer patients without adjuvant treatments (e.g.: adjuvant chemotherapy and endocrine therapy). The median OS of patients with high SLCO1B3 expression and low SLCO1B3 expression were 53.28 and 35 months, respectively (HR: 0.76, 95% CI: 0.65–0.89, *P* < 0.001) (Fig. S2).

#### Univariate and COX multivariate analysis of the influencing factors for DFS and OS of breast cancer patients

Univariate and multivariate COX analysis showed that lymph node metastasis (HR = 3.189 *P* = 0.001), tumor size (HR = 2.543, *P* = 0.007), vascular invasion (HR = 3.128, *P* = 0.033) , PR status (HR = 0.485, *P* = 0.030), menopausal status (HR = 0.474, *P* = 0.021) and Ki-67 index (HR = 3.041, *P* = 0.002) were independent prognostic factors for DFS in breast cancer patients (Table 2). On the other hand, our results indicated that tumor size > 2 cm (HR = 2.291, *P* = 0.014), the presence of vascular invasion (HR = 5.989, *P* = 0.014), lymph node metastasis (HR = 4.515, *P* < 0.001) and histological grade III (HR = 2.564, *P* = 0.009) were significantly associated with worse OS. In contrast, high SLCO1B3 expression (HR = 0.318, *P* = 0.001) was a predictor of favorable OS for breast cancer patients (Table 3).

**Table 2:** Univariate and multivariate analysis of DFS in 315 breast cancer patients.

Variable	Univariate	multivariate	Hazard ratio (95% CI)
	*P* value	*P* value	
Age (> 35 vs. ≤ 35)	0.522		
Menopausal status (Post vs. Pre)	0.042	0.021	0.474 (0.251, 0.893)
Tumor size (≤ 2 vs. > 2 cm)	0.009	0.007	2.543 (1.292, 5.008)
Lymph node metastasis (Yes vs. No)	0.001	0.001	3.189 (1.656, 6.142)
Vascular invasion (Yes vs. No)	0.037	0.033	3.128 (1.099, 8.908)
Histological grade (III vs. I–II)	0.047	0.125	1.749 (0.856, 3.573)
ER status (Positive vs. Negative)	0.453		
PR status (Positive vs. Negative)	0.041	0.030	0.485 (0.253, 0.930)
Ki-67 (≥ 14% vs. < 14%)	0.007	0.002	3.041 (1.521, 6.080)
HER-2 status (Positive vs. Negative)	0.248		
SLCO1B3 expression (Positive vs. Negative)	0.209		
Chemotherapy (Yes vs. No)	0.600		
Endocrine therapy (Yes vs. No)	0.495		

*P* values less than 0.05 are considered as significant changes.

**Table 3:** Univariate and multivariate analysis of OS in 315 breast cancer patients.

Variable	Univariate	Multivariate	Hazard ratio (95% CI)
	*P* value	*P* value	
Age (> 35 vs. ≤ 35)	0.268		
Menopausal status (Post vs. Pre)	0.946		
Tumor size (≤ 2 vs. > 2 cm)	0.011	0.014	2.291 (1.179, 4.453)
Lymph node metastasis (Yes vs. No)	< 0.001	< 0.001	4.515 (2.125, 9.594)
Vascular invasion (Yes vs. No)	0.026	0.014	5.989 (1.432, 25.046)
Histological grade (III vs. I–II)	0.010	0.009	2.564 (1.263, 5.205)
ER status (Positive vs. Negative)	0.327		
PR status (Positive vs. Negative)	0.026	0.337	0.732 (0.387, 1.384)
Ki-67 (≥ 14% vs. < 14%)	0.658		
HER-2 status (Positive vs. Negative)	0.124		
SLCO1B3 expression (Positive vs. Negative)	0.015	0.001	0.318 (0.166, 0.610)
Chemotherapy (Yes vs. No)	0.240		
Endocrine therapy (Yes vs. No)	0.717		

P values less than 0.05 are considered as significant changes.

For ER + breast cancer patients, the results of univariate and multivariate COX analysis demonstrated that lymph node metastasis (HR = 4.079, *P* = 0.001), vascular invasion (HR = 3.603, *P* = 0.040) and PR status (HR = 0.346, *P* = 0.015) were independent predictive factors for DFS (Table 4). In addition, lymph node metastasis (HR = 7.346, *P* < 0.001), vascular invasion (HR = 4.545, *P* = 0.043), histological grade (HR = 3.762, *P* = 0.003), PR status (HR = 0.267, *P* = 0.002) and SLCO1B3 expression (HR = 0.275, *P* = 0.002) were independently associated with the OS of ER + breast cancer patients (Table 5). There were a significant relationship between high SLCO1B3 expression and better OS.

**Table 4:** Univariate and multivariate analysis of DFS in ER + breast cancer patients.

Variable	Univariate	multivariate	Hazard ratio (95% CI)
	*P* value	*P* value	
Age (> 35 vs. ≤ 35)	0.639		
Menopausal status (Post vs. Pre)	0.180		
Tumor size (≤ 2 vs. > 2 cm)	0.031	0.078	2.031 (0.923, 4.471)
Lymph node metastasis (Yes vs. No)	0.001	0.001	4.079 (1.741, 9.558)
Vascular invasion (Yes vs. No)	0.049	0.040	3.603 (1.061, 12.235)
Histological grade (III vs. I–II)	0.116		
PR status (Positive vs. Negative)	0.017	0.015	0.346 (0.147, 0.813)
Ki-67 (≥ 14% vs. < 14%)	0.319		
HER-2 status (Positive vs. Negative)	0.367		
SLCO1B3 expression (Positive vs. Negative)	0.352		
Chemotherapy (Yes vs. No)	0.611		
Endocrine therapy (Yes vs. No)	0.737		

*P* values less than 0.05 are considered as significant changes.

**Table 5:** Univariate and multivariate analysis of OS in ER + breast cancer patients.

Variable	Univariate	Multivariate	Hazard ratio (95% CI)
	*P* value	*P* value	
Age (> 35 vs. ≤ 35)	0.333		
Menopausal status (Post vs. Pre)	0.758		
Tumor size (≤ 2 vs. > 2 cm)	0.036	0.217	1.686 (0.735, 3.868)
Lymph node metastasis (Yes vs. No)	0.001	< 0.001	7.346 (2.654, 20.335)
Vascular invasion (Yes vs. No)	0.042	0.043	4.545 (1.051, 19.662)
Histological grade (III vs. I–II)	0.008	0.003	3.762 (1.567, 9.030)
PR status (Positive vs. Negative)	0.002	0.002	0.267 (0.114, 0.624)
Ki-67 (≥ 14% vs. < 14%)	0.949		
HER-2 status (Positive vs. Negative)	0.313		
SLCO1B3 expression (Positive vs. Negative)	0.046	0.002	0.275 (0.120, 0.627)
Chemotherapy (Yes vs. No)	0.577		
Endocrine therapy (Yes vs. No)	0.586		

*P* values less than 0.05 are considered as significant changes.

### mRNA level of SLCO1B3 in MCF-10A, MDA-MB-231, BT-549, MCF-7 and MDA-MB-453 cell lines

**Figure 2:**
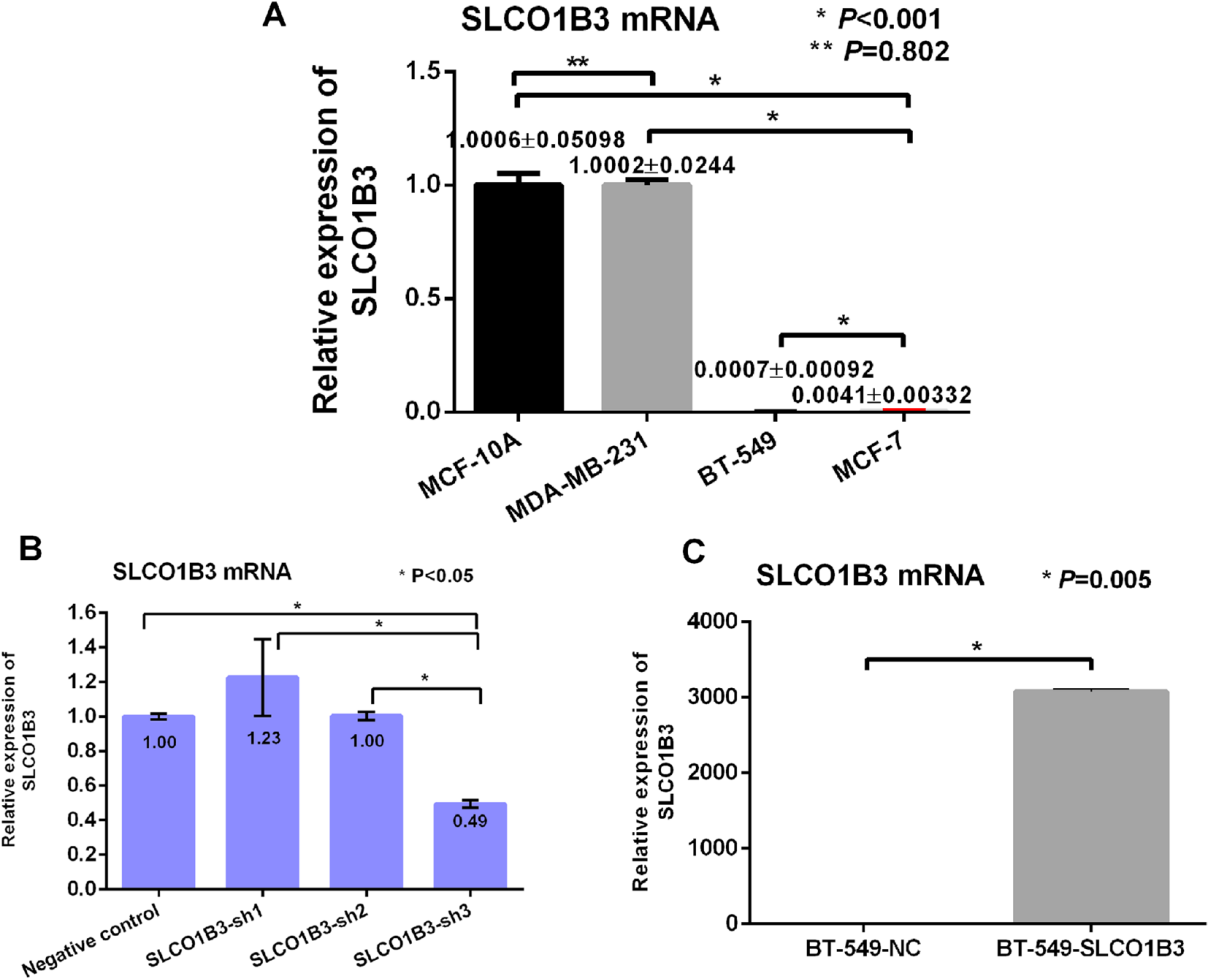
A. The mRNA level of SLCO1B3 in normal breast cells and different breast cancer cell lines. B. The mRNA level of SLCO1B3 in the transfected MDA-MB-231 cells. C. The mRNA level of SLCO1B3 in BT-549 cells after SLCO1B3 overexpression.

SLCO1B3 mRNA was highly expressed in normal breast cells MCF-10A. Among four different cell lines, the relative expression level of SLCO1B3 in MDA-MB-453 cell line was not stable. The relative expression level of SLCO1B3 mRNA in MDA-MB-231 was highest among the tested cell lines, and BT-549 cell line had the lowest relative expression level for SLCO1B3 mRNA (*P* < 0.001) (Fig. 2A). MDA-MB-231 breast cancer cells were used for knockdown experiment (Fig. 2B), and BT-549 breast cancer cells were selected for overexpression experiment (Fig. 2C).

### Determination of cell migration and invasion ability by Transwell assay

**Figure 3:**
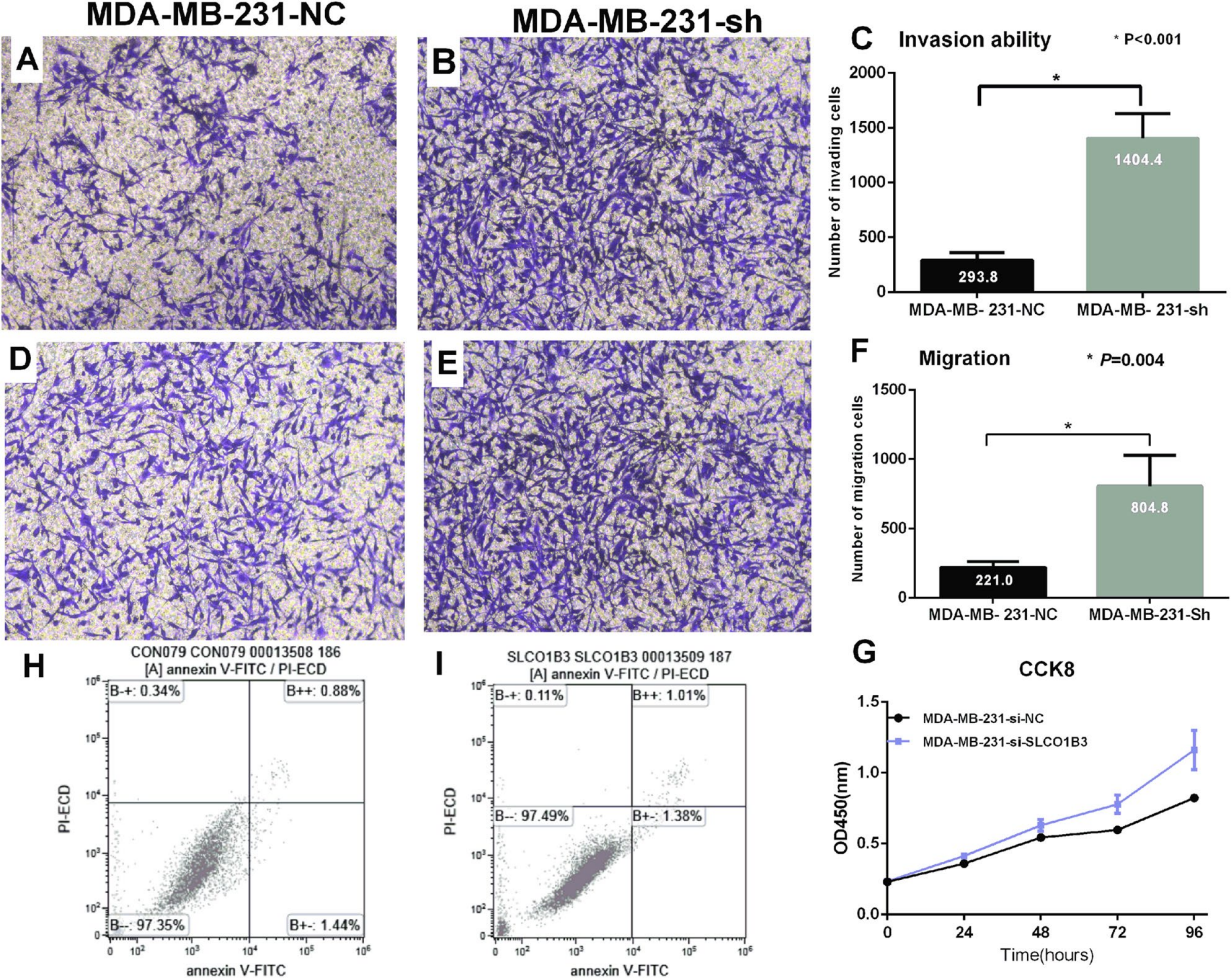
Evaluation of the MDA-MB-231 cell invasion, migration, proliferation and apoptotic ability after knockdown of SLCO1B3. (**A**,**B**) The number of cells migrated to the lower chamber in the empty plasmid control group and SLCO1B3 knockdown group. (**C**) Comparison of the invasion ability between the two groups. (**D**,**E**). The number of cells migrated to the lower chamber in the empty plasmid control group and SLCO1B3 knockdown group. (**F**) Comparison of the migration ability between the two groups. (**H**,**I**) The apoptotic ability of the empty plasmid control group and SLCO1B3 knockdown group. (**G**) Comparison of the proliferation ability between the empty plasmid control group and SLCO1B3 knockdown group.

**Figure 4:**
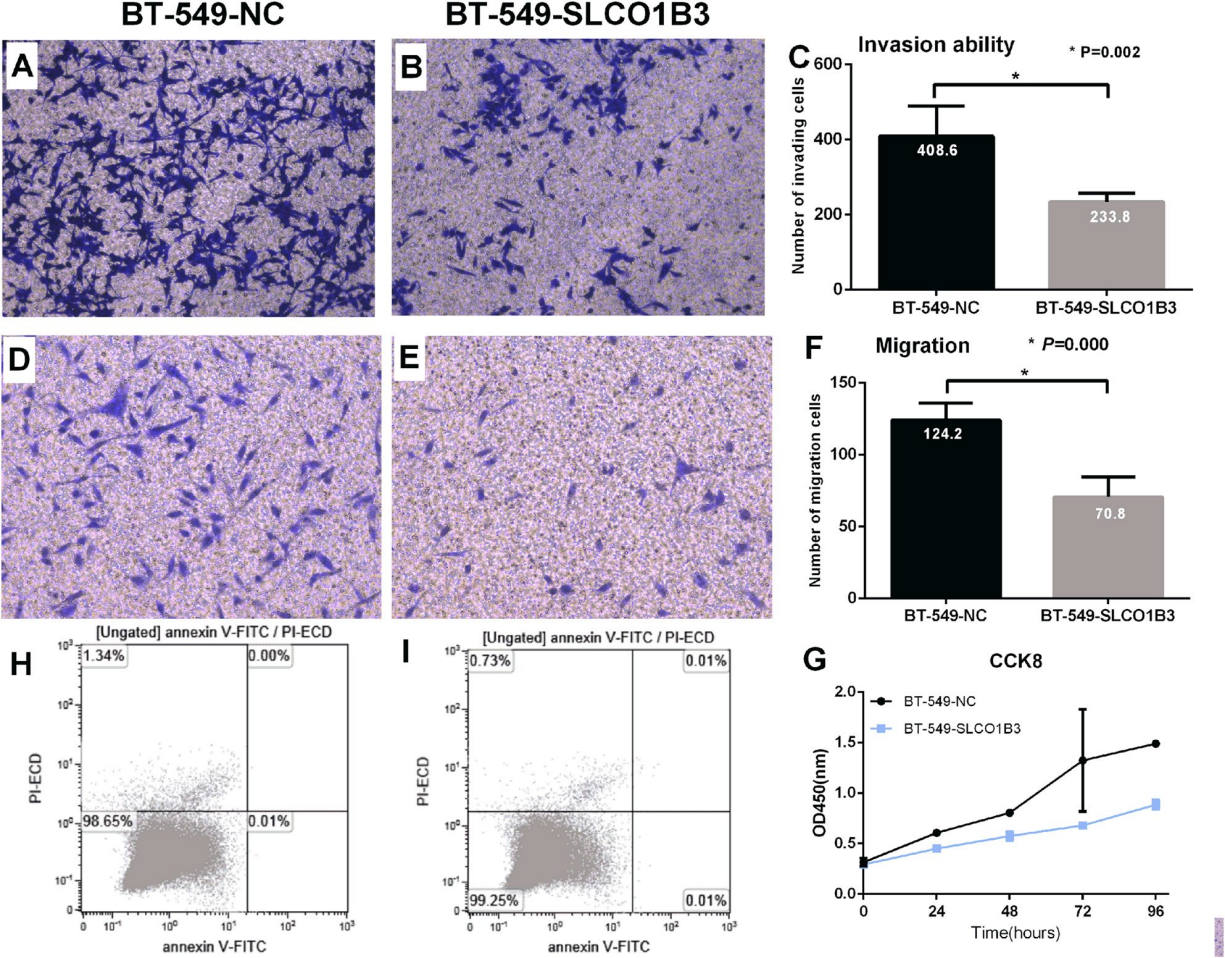
Evaluation of the BT-549 cell invasion, migration, proliferation and apoptotic ability after SLCO1B3 overexpression. (**A**,**B**) The number of cells migrated to the lower chamber in the empty plasmid control group and SLCO1B3 overexpression group. (**C**) Comparison of the invasion ability between the two groups. (**D**,**E**) The number of cells migrated to the lower chamber in the empty plasmid control group and SLCO1B3 overexpression group. (**F**) Comparison of the migration ability between the two groups. (**H**,**I**) The apoptotic ability of the empty plasmid control group and SLCO1B3 overexpression group. (**G**) Comparison of the proliferation ability between the empty plasmid control group and SLCO1B3 overexpression group.

Transwell assay was used to evaluate the invasion and migration ability of the cells after overexpression or knockdown of SLCO1B3 in tested cell lines. MDA-MB-231 cells with knockdown of SLCO1B3 (MDA-MB-231-Sh) exhibited increased ability of penetrating membrane compared to the negative control group (MDA-MB-231-NC) (*P* < 0.001) (Fig. 3A–F). In contrast, overexpression of SLCO1B3 in BT-549 cells resulted in a reduced ability of invasion and migration of membrane compared to the negative control cells (*P* < 0.001) (Fig. 4A–F).

### The effect of overexpression or knockdown of SLCO1B3 on cell proliferation

The result from CCK8 assay showed that the proliferation ability of MDA-MB-231 cells was significantly increased after knocking down SLCO1B3 (*P* < 0.001) (Fig. 3G). In contrast, the proliferation ability of BT-549 cells was significantly reduced after overexpression of SLCO1B3 (*P* < 0.001) (Fig. 4G).

### Detection of apoptosis by flow cytometry

The flow cytometry assay indicated that overexpression or knockdown of SLCO1B3 in breast cancer cells had no effect on apoptotic ability of cells. (MDA-MB-231-NC vs. MDA-MB-231-Sh, 0.02% vs. 0.01%, *P* = 0.068; BT-549-NC vs. BT-549-SLCO1B3, 2.32% vs. 2.39%, *P* = 0.287) (Figs. 3H, I; 4H, I).

## Discussion

SLCO1B3 gene is located on 12p12-31.7 to 12p12-37.2 of human chromosomes, and encodes a transmembrane protein consisting of 702 residues^10^. SLCO1B3 consists of 12 intracellular N- and C-terminal predicted transmembrane domains based on hydrophobicity analysis^11^, and it is a liver-specific transporter. The expression of SLCO1B3 and its functional change have been detected in a variety of malignant tumors. However, the molecular regulatory mechanism underlying this process in tumor tissues is not well known^12–14^.

The studies about SLCO1B3 expression in normal tissues showed different findings. Some studies reported that SLCO1B3 was not expressed in normal colon cancer tissues^5^ or normal breast tissues^8^. But in normal hepatocytes, SLCO1B3 is highly expressed, and 60% of liver cancer tissues have decreased SLCO1B3 expression compared to normal adjacent tissues^15^. Until now, there had no report about the positive expression of SLCO1B3 in normal breast tissues, other members in the same family, such as OATP2B1, OATP3A1 and OATP5A1, were found to be highly expressed in normal breast tissues^9^. However, in this study, SLCO1B3 was not only highly expressed on the cell membrane and cytoplasm of normal breast tissues, but was also expressed on the same locations of breast cancer tissues. Our study showed that 80% of breast cancer tissues were positive for SLCO1B3 expression, and 24% of them were strongly positive. Moreover, the patients with strong SLCO1B3 expression tended to have negative lymph node metastasis, AJCC stage I, and histological grade 1 (*P* < 0.05), while in the SLCO1B3 negative patients, there were more cases with positive lymph node metastasis, especially with the number of metastases ≥ 3, and the percentage of patients with histological grade 3 breast cancer was higher (*P* < 0.05). Survival analysis showed that although DFS was not significantly different among the three SLCO1B3 expression groups (*P* = 0.016), the strongly positive group had a significantly improved DFS compared to the negative group (*P* = 0.042). Similarly, for OS, the survival rate of strongly positive group was increased (*P* = 0.000), especially when compared with the SLCO1B3 negative group (*P* = 0.001), suggesting that the higher the expression level of SLCO1B3, the lower the proportions of recurrence, metastasis and death. These results are consistent with the findings from Muto et al.^8^, indicating that SLCO1B3 is a good prognostic factor for breast cancer. Unlike Muto’s report, our study showed that the higher the expression level of SLCO1B3, the more obvious the difference.

COX multivariate analysis showed that lymph node metastasis, tumor size, vascular invasion, PR, and menstrual status were independent prognostic factors for DFS, which is consistent with other studies. But SLCO1B3 expression is not correlated with DFS. The same COX multivariate analysis was also performed for OS, and more lymph node metastasis (HR = 2.991, *P* = 0.000), larger tumor size (HR = 2.075, *P* = 0.003), and vascular invasion (HR = 6.759, *P* = 0.009) could increase the risk of death; while higher SLCO1B3 expression (HR = 0.574, *P* = 0.018) and positive PR (HR = 0.369, *P* = 0.001) could reduce the risk of death, which were independent prognostic factor for OS.

Previous studies have shown that the uptake of estradiol-3-sulfate in T47-D breast cancer cells could lead to increased cell proliferation via the Na^+^ transport system, and the uptake of estradiol-3-sulfate was mediated by OATP^16,17^, suggesting that OATP might promote the proliferation of breast cancer cells. However, Muto et al.^8^ showed that the expression of SLCO1B3 was negatively correlated with tumor size, and was significantly correlated with reduced recurrence and good prognosis. Importantly, this association only appeared in postmenopausal women. Thus, these results suggested that SLCO1B3 could transport estradiol-3-sulfate, and its overexpression was related to the hormone-dependent growth mechanism of breast cancer^8^. In our study, we found that in the ER positive patients, the stronger the expression level of SLCO1B3, the better the patient OS (*P* = 0.002). Although there was no significant difference in DFS between the three groups, the strongly SLCO1B3 positive group exhibited better trend of DFS than the negative group (*P* = 0.042). On the other hand, in ER negative patients, there was no association between DFS/OS and SLCO1B3 expression (*P* = 0.926, *P* = 0.531). These results suggested that the expression of SLCO1B3 and its effect on breast cancer were related to estrogen.

The result from in vitro CCK-8 assay indicated that knockdown of SLCO1B3 expression promoted the proliferation of breast cancer cell MDA-MB-231, and the difference was statistically significant (*P* < 0.001). In contrast, overexpression of SLCO1B3 in BT-549 resulted in significant reduction of cell proliferation (*P* < 0.001), suggesting that SLCO1B3 plays an important role in cell proliferation. In addition, we found that knockdown of SLCO1B3 led to an increased ability for MDA-MB-231 cells to penetrate the membrane, whereas overexpression of SLCO1B3 in BT-549 resulted in decreased cell number penetrating membrane, suggesting that SLCO1B3 is also involved in the invasion and migration of breast cancer cells, and the expression of SLCO1B3 can inhibit the migration and invasion of breast cancer cells. However, we did not observe the effect of SLCO1B3 for the apoptotic ability of breast cancer cells.

It is possible that the role of SLCO1B3 in tumor tissues, especially in hormone-dependent tumors, is to promote cancer progression by stimulating the production of intracellular hormones, or it may influence the prognosis of tumor patients by affecting the absorption of therapeutic drugs through some mechanisms^18–21^. In endometrial cancer, it was shown that the higher the expression level of SLCO1B3, the longer the disease-free survival time and overall survival time^22^. These studies indicated that SLCO1B3 acts as a suppressor in estrogen-related tumors, which is also consistent with the results from this study.

In addition, the results obtained from the in vitro assay in this study were also consistent with the results from clinical study. The high expression of SLCO1B3 in breast cancer tissues was negatively correlated with breast cancer stage, lymph node metastasis status, and histological grade. In addition, the higher the expression of SLCO1B3, the longer the overall survival rate of patients. The in vitro study indicated that SLCO1B3 may be involved in the proliferation, migration and metastasis of tumor cells, and may play a role in suppressing tumor during the progression of breast cancer. SLCO1B3 may serve a potential biomarker for breast cancer diagnosis, treatment and prognosis evaluation.

## Limitation

The current study is a retrospective study. Due to some missing data, the section of patients might be biased, which could affect the analytic results. Moreover, we could not evaluate the impact of SLCO1B3 expression on cancer-specific survival (CSS) in breast cancer patients since the relevant data was scarce. In this study, the results showed that SLCO1B3 high expression was significantly associated with the absence of lymph node metastasis, low histological grade and TNM stage. The favorable survival in patients with SLCO1B3 high expression may be attributed to these factors. However, we also performed the multivariate COX analysis to adjust all potential covariates, and the results strengthened the prognostic significance of SLCO1B3 expression for breast cancer patients. In addition, the mechanism by which SLCO1B3 inhibits the occurrence and development of breast cancer is still unclear, which needs to be further explored.

## Conclusions

By immunohistochemistry, the higher the expression of SLCO1B3, the lower the proportion of recurrence, metastasis and death. In the ER positive patients, there was a significant association between DFS/OS and SLCO1B3 expression. Therefore, SLCO1B3 is a potential indicator for breast cancer prognosis, and its function may be related to estrogen status in breast cancer. In vitro study indicated that SLCO1B3 can inhibit the proliferation, migration, and invasion of breast cancer cells, suggesting that SLCO1B3 may play a suppressive role in the development of breast cancer. Our study provided basis for further studies, and highlighted the usefulness of SLCO1B3 as a potential biomarker for diagnosis, treatment, and prognosis evaluation in breast cancer. Further studies are needed to characterize the mechanism by which SLCO1B3 regulates the development of breast cancer.

## Materials and methods

### Study population

The paraffin-embedded tissues were collected from 357 patients with primary invasive breast cancer who were admitted to the Breast Center of the 4th Hospital of Hebei Medical University from January 1, 2012 to December 31, 2012. The present study was approved by the independent ethics committee of the Fourth Hospital of Hebei Medical University. Informed consent was obtained from all participants. All specimens were obtained under the protocols outlined by the institutional review board. All experiments were performed in accordance with relevant guidelines and regulations. The breast cancer was confirmed by pathology, and the follow-up information for these patients were intact. Inoperable patients, and the patients with stage IV and ductal carcinoma in situ were excluded. After all specimens were stained, 42 cases were excluded due to poor staining, and a total of 315 patients were included in the final analysis. We grouped the main follow-up results based on SLCO1B3 expression levels, and compared the disease-free survival (DFS) and overall survival (OS) among different groups. DFS was defined as the time since the day of surgery until the first occurrence of recurrent disease at any site. OS was defined as the time from diagnosis to death from any cause. All patients were re-examined every 4–6 months within 2 years after operation, followed by every 6 months within 5 years, and then once a year after 5 years. All patients were followed up by telephone or outpatient clinic.

### Immunohistochemistry

Serial sections were obtained from the paraffin blocks, and the two-step immunohistochemistry SP method was used to stain the sections. The slides were developed with DAB, and the expression level of SLCO1B3 was determined under a microscope. Rabbit polyclonal antibody for SLCO1B3 (1:200) was raised against the synthetic carboxyl-terminal peptide of human SLCO1B3, the secondary antibody was raised against the synthetic NH2-terminal peptide of HRP conjugated goat anti-mouse/rabbit IgG antibody.

### Determination of the cut-off point of SLCO1B3 expression

SLCO1B3 staining was positive for both cell membrane and cytoplasm, but cell membrane had the main staining. The immunohistochemistry scoring standard was based on both staining intensity and the ratio of positive cells. The specific criteria were as follows: (1). Ratio of positive cells: five high-magnification fields were randomly selected in each section, and the number of positively stained cells was counted: 1 point, 1–24%; 2 points, 25–49%; 3 points, 50–74%; 4 points, ≥ 75%. (2). Staining intensity: 0 point, no staining; 1 point, weak staining; 2 points, moderate staining; 3 points, strong staining. In order to evaluate the prognostic value of SLCO1B3 expression level, we used different cut-off points to verify the relationship between SLCO1B3 expression and DFS/OS. The final grouping criteria were based on the sum of staining intensity score and the ratio of positive cell score: negative, < 2; moderate positive, 3–5; strongly positive, 6–7 (Fig. 1A–C). All the other grouping criteria, such as negative and positive, intensity alone, or positive cell ratio alone, did not show significant difference. In the 315 patients, 62 cases showed negative SLCO1B3 expression (19.7%), 177 cases had moderate expression (56.2%), and 76 cases showed strong expression (24.1%).

The IHC staining for surgical samples of breast cancer was performed by Ventana-automated machine. The primary monoclonal mouse antihuman estrogen receptor (Roche Company), monoclonal mouse antihuman progesterone receptor (Roche Company), monoclonal mouse anti-human c-erbB-2 oncoprotein (Roche Company) and anti-Ki67 (Roche Company) monoclonal rabbit antibody were used to evaluate the expression of ER, PR, HER2 and Ki67, respectively.

Positive hormone receptor (HR) expression was defined as the presence of 10% staining of any intensity for either estrogen receptor (ER) or progesterone receptor (PR). Patients with “low-positive” staining for ER or PR, as defined by the current ASCO/CAP (1–9% staining of any intensity), were considered to have negative HR expression. On the other hand, HER-2 expression was determined by immunohistochemical staining (IHC) or fluorescence in situ hybridization (FISH), and HER-2 positivity was described when membrane staining was scored 2 + by IHC or HER-2 gene amplification was detected by FISH. In the present study, Ki-67 index was defined according to the St Gallen recommendations, and all patients were divided into Ki-67 low expression (< 14%) and high expression (≥ 14%) group. The molecular subtypes of breast cancer were classified by the St Gallen Consensus (2013): Luminal A type (ER + and/or PR + , HER2- and Ki67% < 14%), Luminal B type (Luminal B, HER2- type: ER + and/or PR + , HER2- and Ki67% ≥ 14%; Luminal B, HER2 + type: ER + and/or PR + , HER2 + and Ki67% ≥ 14%), HER2 type (ER-, PR-, and HER2 +), and triple negative type (ER-, PR-, and HER2-).

### Cell lines, reagents and instruments

Human normal breast cell line MCF-10A, breast cancer cell lines MDA-MB-231 (triple negative type), MDA-MB-453 (ER-, PR- and HER2 + , which represented HER2 + type), BT-549 (triple negative type), and MCF-7 (ER + , PR + and HER2-, which was similar to luminal A type) were purchased from the cell bank of Chinese Academy of Medical Sciences. All cell lines were maintained in RPMI1640 medium with 10% fetal bovine serum (FBS), and supplemented with 100 U/L streptomycin and penicillin, and cultured in a cell incubator containing 5% CO_2_ at 37 °C. The cells were passaged every 2–3 days.

RPMI1640 medium and FBS were purchased from Gibco (Life Technologies, Paisley, Auckland). Lipofectamine 2000, general RNA isolation kit, and Trizol were purchased from Invitrogen (Carlsbad, CA, USA). RNA reverse transcription kit, qPCR kit, and Go Taq qPCR Master Mix were purchased from Promega (Madison, Wisconsin, USA). Matrigel matrix was purchased from BD (San Diego, CA, USA). Primers were obtained from Sangon Biotech (Shanghai, China). SLCO1B3 knockout kit was obtained from GENE (Shanghai, China). Transwell plate was obtained from Corning (Tewksbury, MA, USA). CCK-8 kit was obtained from Solarbio (Beijing, China), and fluorescence real-time PCR instrument were obtained from Thermo (Waltham, MA, USA).

### Detection of SLCO1B3 mRNA level in MCF-10A, MDA-MB-231, BT-549, MCF7 and MDA-MB-453 cell lines by qRT-PCR

MCF-10A, MDA-MB-231, BT-549, MCF-7, and MDA-MB-453 cells grown in logarithmic growth phase with good growth condition were harvested and the general RNA from each cell line was extracted using general RNA isolation kit according to the instruction. cDNA was synthesized with reverse transcription by using general RNA as template. The synthesized cDNA was then used as template to perform SYBR-Green I-based qRT-PCR. Human GAPDH was amplified as a housekeeping gene to normalize the data. The qRT-PCR was performed with a standard procedure and each sample was tested in triplicate. The reaction was performed as following condition: Initial denaturation at 95 °C for 3 min, followed by 40 cycles of denaturation at 95 °C for 12 s, and annealing at 62 °C for 40 s. The primers included SLCO1B3-forward: 5′-TCATTGGCTTTGCACTGGGA-3, SLCO1B3-reverse: 3′-AAACCAAGCCACCAAGCTCC-5′, and the internal control gene GAPDH-forward: 5′-AGCCACATCGCTCAGACAC-3′, and GAPDH reverse: 3′-GCCCAATACGACCAAATCC-5′ were used in the study. The relative expression level of SLCO1B3 was calculated with 2^−△△CT^method, △CT = CT_SLCO1B3_ − CT_GAPDH_.

Breast cancer cells with low expression of SLCO1B3 were transfected with overexpression plasmids, while breast cancer cells with high expression of SLCO1B3 were transfected with knockdown plasmids. In addition, the two cell lines were also transfected with empty plasmids, respectively.

*Reagent 1* 6 μl Lipofectamine 2000 was mixed with 244 µl FBS- and antibiotic-free RPMI1640 medium for 5 min at room temperature.

*Reagent 2* 10 μl shRNA plasmid SLCO1B3-sh1-3, SLCO1B3 overexpression plasmid, or control plasmid (4 μg for each) was mixed with 240 µl FBS- and antibiotic-free RPMI1640 medium for 5 min at room temperature.

The breast cancer cells with low expression of SLCO1B3 and high expression of SLCO1B3 in the logarithmic growth phase were harvested and inoculated into six-well plates respectively. When the cell confluence reached 70%-80%, the overexpression plasmid, knockdown plasmid, or empty plasmid was transfected to breast cancer cells with low expression of SLCO1B3 or cells with high expression level of SLCO1B3, respectively, according to the instruction of Lipofectamine 2000. The transfected cells were harvested and washed twice with PBS buffer at 48 h after transfection. The mRNA level of SLCO1B3 in cells were detected by qRT-PCR and quantified using 2^-△△CT^ method. The knockdown experiment was repeated for three times. The plasmids with best inhibitory effect or overexpression effect were selected for further experiments.

After 48 h transfection of cells with different plasmids, two different cell lines were diluted with RPMI1640 culture medium at a ratio of 1: 10, and the cells were added to 6-well plate with 5 × 10^4^ cells/well, followed by adding screening concentration of puromycin (600 ng/ml). After 14 days of culture, the surviving cells were stably transfected cells.

### Cell proliferation assay

The cells successfully transfected with SLCO1B3 overexpression plasmid, SLCO1B3 knockdown plasmid, or empty plasmid control were seeded into 96-well plate with a cell density of 1.0 × 10^3^ cells/well, and six wells per group. The cells were then incubated in a cell incubator containing 5% CO_2_ at 37 °C. 10 µl CCK-8 was added to each well at 0, 24, 48, 72, and 96 h after cell attachment and gently mixed, followed by incubation for 1–2 h in the cell incubator. The absorbance value of cells in each well at 450 nm wavelength was measured by CCK8 method using a microplate reader. The experiment was repeated three times.

### Determination of cell invasion and migration ability by Transwell assay

The cells with overexpression of SLCO1B3, knockdown of SLCO1B3, or transfected with empty plasmid grown in logarithmic growth phase with good growth condition were incubated for 12 h with serum starvation. After digestion, washing, and centrifugation, during the migration experiment, the cells were resuspended in serum-free RPMI-1640 medium. 200 μl of cell suspensions containing 2 × 10^4^ cells were transferred into the upper chamber of Transwell, and the lower chamber of Transwell was filled with 600 μl complete RPMI-1640 medium. The plate was then incubated incubate for 24 h in a 5% CO_2_ incubator at 37 °C. The upper chamber was removed and the cells in upper chamber were wiped off with a cotton swab. Cells that migrated to the lower chamber were stained with crystal violet and counted under a 100 × microscopy (5 fields were randomly selected). However, during the invasion experiment, Matrigel and serum-free RPMI1640 were diluted 1:8 and coated with the upper side of the basement membrane of the Transwell and 60 µl of diluted Matrigel was added to each hole. The remaining steps are the same as the migration experiment.

### Apoptosis analysis by flow cytometry

The different groups of cells, including empty plasmid control groups MDA-MB-231-NC and BT-549-NC, SLCO1B3 knockdown group MDA-MB-231-Sh, and SLCO1B3 overexpression group cells BT-549-SLCO1B3, with good growth in logarithmic growth phase, were harvested and washed twice with cold PBS, and the cell density was adjusted to 1 × 10^7^/ml. 100 μl cell suspension of each group was collected and the cells were washed once with cold PBS. The cells were pelleted by centrifugation and resuspended in 100 μl cold 1 × binding buffer, followed by adding 10 μl Annexin V-FITC and PI. The cells were incubated on ice without light for 15 min, then 380 μl 1 × binding buffer was added, followed by adding 10 μl 7-AAD staining solution, and incubated the cells on ice for 15 min in the dark. The cells were washed once with cold PBS, and then resuspended with 1 ml PBS. The cell samples were tested on a flow cytometer.

### Statistical analysis

The statistical analysis was performed by using SPSS 13.0 software (IBM Inc, New York, USA) and Graphpad Prism 5. The data was expressed as Mean ± SD. The counting data were expressed in median (interquartile range), and Mantel-Haenszed Chi-squared test was used for comparison and correlation. Kaplan–Meier method was used to analyze DFS and OS, and Log-rank test was used to compare the trends between two groups. Multivariate survival analysis was performed using the COX risk regression model. The analysis of variance was used for comparing three or more groups, and further comparison between groups was performed with LSD-t test. T test was used for comparison of two groups. A *P* value smaller than 0.05 was considered statistically significant.

## Supplementary Information

Supplementary Information.

## Data Availability

All data generated or analyzed during this study are included in this article.
