# Supplementary material for: Highly expressed SLCO1B3 inhibits the occurrence and development of breast cancer and can be used as a clinical indicator of prognosis
Source: Sci Rep. 2021 Jan 12;11:631. doi: 10.1038/s41598-020-80152-0 (PMC7803962; doi:10.1038/s41598-020-80152-0)

**Highly expressed SLCO1B3 inhibits the occurrence and development of breast cancer and can be used as a clinical indicator of prognosis**

Tiantian Tang^1^, Guiying Wang^2,3*^, Sihua Liu^4^, Zhaoxue Zhang^1^, Chen Liu^1^, Fang Li^5^, Xudi Liu^5^, Lingjiao Meng^4^, Huichai Yang^5^, Chunxiao Li^1^, Meixiang Sang^4^, Lianmei Zhao^4^

^1^ Breast Cancer Center, the Fourth Hospital of Hebei Medical University, Shijiazhuang, Hebei Province 050035, China;

^2^ Department of [General](C:/Users/TTT/AppData/Local/youdao/dict/Application/8.7.0.0/resultui/html/index.html#/javascript:;) Surgery, the Fourth Hospital of Hebei Medical University, Shijiazhuang, Hebei Province 050035, China;

^3^ Department of General Surgery, the Third Hospital of Hebei Medical University, Shijiazhuang, Hebei Province 050001, China;

^4^ Scientific Research Center, the Fourth Hospital of Hebei Medical University, Shijiazhuang, Hebei Province 050035, China;

^5^ Department of Pathology, the Fourth Hospital of Hebei Medical University, Shijiazhuang, Hebei Province 050035, China;

***Corresponding Author:** Guiying Wang, M.D. Department of General Surgery, the Fourth Hospital of Hebei Medical University, Shijiazhuang, Hebei Province 050035, China; Department of General Surgery, the Third Hospital of Hebei Medical University, Shijiazhuang, Hebei Province 050001, China

**Tel:** +86 13932186739

**E-mail:** wangguiyingtgzy@163.com

**Running title:** The value of SLCO1B3 in breast cancer

**Figure S1 Comparison of survival outcomes between SLCO1B3 high and low expression in breast cancer patients using the Kaplan-Meier plotter database.**


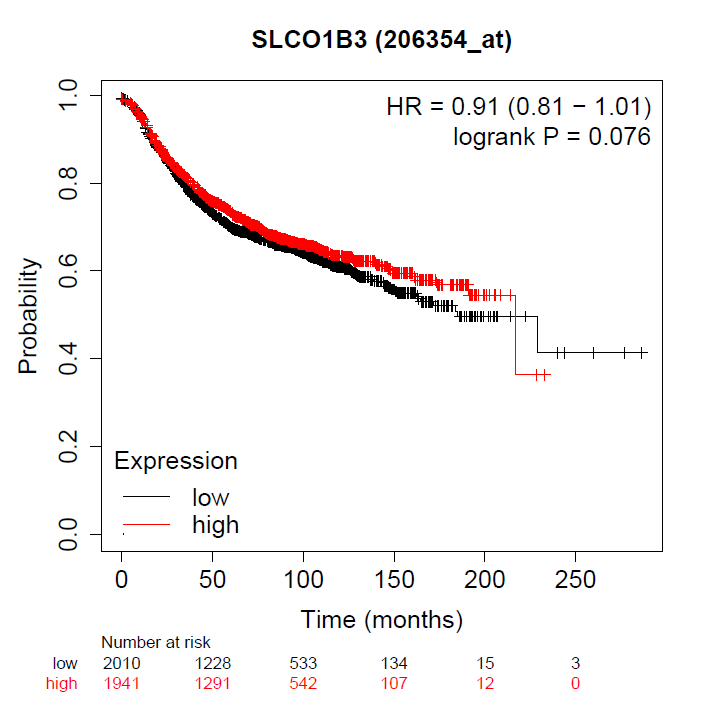


**Figure S2 Comparison of survival outcomes between SLCO1B3 high and low expression in breast cancer patients with adjuvant treatments using the Kaplan-Meier plotter database.**


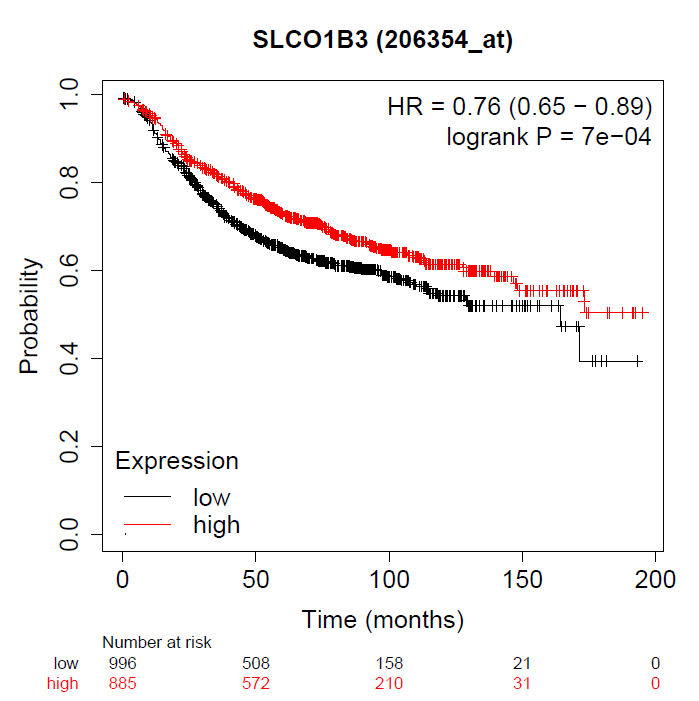

Supplement: Supplementary file 1 — Supplementary Information. [file 41598_2020_80152_MOESM1_ESM.docx]
